# The possible anti-tumor actions and mechanisms of active metabolites from Cortex Fraxini

**DOI:** 10.3389/fphar.2024.1404172

**Published:** 2024-09-13

**Authors:** Bin Cai, Ting Cai, Zeyu Feng, Huanhuan Zhu

**Affiliations:** ^1^ Department of Anorectal Surgery, Wuxi Hospital Affiliated to Nanjing University of Chinese Medicine, Wuxi, China; ^2^ Department of Nephrology, The Affiliated Wuxi People’s Hospital of Nanjing Medical University, Wuxi People’s Hospital, Wuxi Medical Center, Wuxi, China

**Keywords:** Cortex Fraxini, anti-tumor, esculin, esculetin, fraxetin

## Abstract

Cortex Fraxini is a traditional Chinese herb that is widely available, inexpensive, and has low toxicity. Modern pharmacological studies have demonstrated that the active metabolites in Cortex Fraxini, including esculin, esculetin, and fraxetin, exert anti-tumor activities by regulating genes and proteins involved in cancer cell proliferation, apoptosis, invasion, and migration. Additionally, these metabolites play a pivotal role in the regulation of several tumor-associated signaling pathways, including the PI3K/Akt, MAPK/ERK, JAK/STAT3, and Wnt/β-catenin pathways. Due to their pro-apoptotic and anti-proliferative properties *in vitro* and *in vivo*, Cortex Fraxini and its active metabolites may be considered as potential candidates for the treatment of tumor. The aim of this review is to highlight the anti-tumor biological activities and underlying mechanisms of action of the active metabolites of Cortex Fraxini, with a view to providing a reference for their further development and application in the treatment of tumors.

## 1 Introduction

**FIGURE 1:**
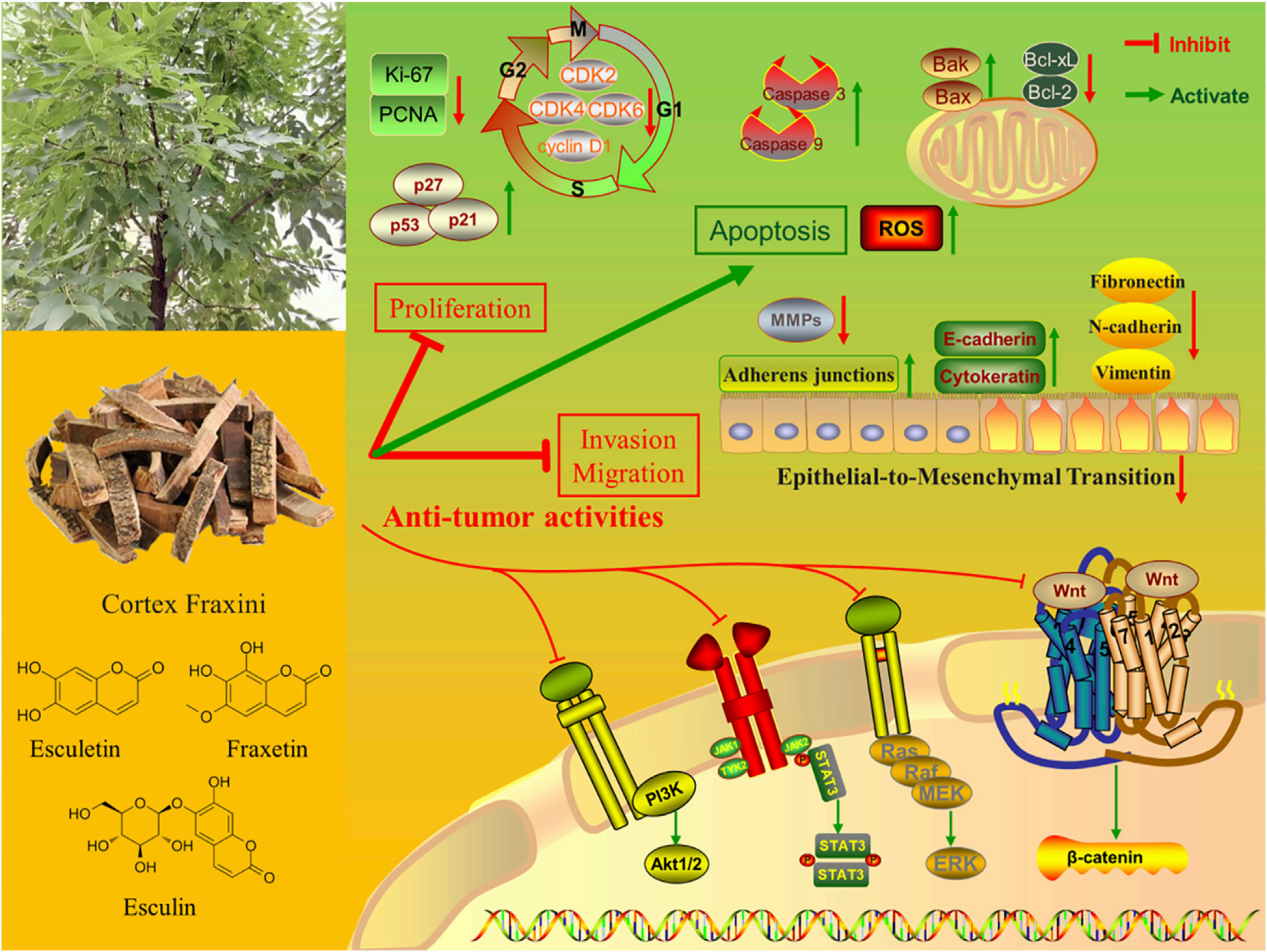
The anti-tumor pharmaceutical activities of Cortex Fraxini and its active metabolites.

Cortex Fraxini (also known as Qinpi in Chinese) is a widely used herbal medicine that belongs to the “heat-clearing” category in traditional Chinese medicine. Cortex Fraxini is derived from the dried bark of *Fraxinus rhynchophylla* Hance, *Fraxinus chinensis* Roxb., *Fraxinus szaboana* Lingelsh., and *Fraxinus stylosa* Lingelsh. As a well-known traditional Chinese herbal medicine, Cortex Fraxini has been used for more than 2,000 years to treat conjunctivitis, diarrhea, hyperuricemia, bacillary dysentery, and excessive leukorrhea (Li et al., 2015; Wang G. et al., 2017). Modern pharmacological studies of Cortex Fraxini have revealed that it has a range of pharmacological effects, including anti-inflammatory, antioxidant, and antibacterial properties. (Wu et al., 2007). To date, phytochemical investigations have identified a variety of components in Cortex Fraxini, mainly coumarins, lignans, secoiridoids, phenolic acids, flavonoids, phenols, triterpenoids, and steroids (Zheng et al., 2024). Pharmacological studies have shown that coumarins are the primary active metabolites of Cortex Fraxini, including esculin, esculetin, fraxin, and fraxetin (Li C. X. et al., 2022). Moreover, numerous *in vivo* and *in vitro* studies have demonstrated that these active metabolites of Cortex Fraxini exert anti-tumor effects through cell cycle regulation, inhibition of tumor cell proliferation, induction of apoptosis, and other mechanisms (Li Z. Y. et al., 2022; Zhang et al., 2022). The anti-tumor effects of these active metabolites have recently attracted the attention of researchers, leading to increased interest in the anti-tumor pharmacological activity of Cortex Fraxini (Figure 1).

Esculin is a glycosidic coumarin derivative (6-beta-glucoside-7-hydroxycoumarin, molecular formula: C15H16O9). The two parts of the molecule (glucose and 7-hydroxycoumarin) are linked by an ester linkage through oxygen. Esculin can be enzymatically hydrolyzed at the 8-glucose linkage to yield two products, esculetin and glucose. Studies have demonstrated that esculin can be distributed throughout the body in a rapid and uniform manner (Li et al., 2013). However, the first-pass effect of esculin was so severe that the calculated oral bioavailability was only 0.62% (Rehman et al., 2015). Esculetin is one of the simplest coumarins with two hydroxyl groups at carbons 6 and 7 (6,7-dihydroxy-2-chromenone, molecular formula: C9H6O4), which is the aglycone metabolite of esculin (Wang et al., 2016). The average oral bioavailability of esculetin was found to be 19%, which was significantly higher than that of esculin (Kwak et al., 2021). Moreover, esculetin demonstrated little cytotoxicity to human normal cells. A limited impact of esculetin on the viability of normal human gastric epithelial cells was observed in an *in vitro* study (Wang Y. et al., 2017). Fraxetin is a simple hydroxycoumarin (7,8-dihydroxy-6-methoxycoumarin, molecular formula: C10H8O5), and has minimal cytotoxic effects on normal human cells. A report on human melanoma demonstrated that high concentrations of fraxetin only slightly inhibited the viability of the human keratinocyte and melanocyte cell lines (Wróblewska-Łuczka et al., 2022). The content of fraxetin in the Cortex Fraxini was lower than that of esculin and esculetin (Zhou et al., 2008). This paper presents a systematic review of the existing studies on the anti-tumor effects of Cortex Fraxini and its active metabolites. Furthermore, our review is of great significance in exploring the anti-tumor potential of Cortex Fraxini, which may provide a reference for further research on Cortex Fraxini and its active metabolites in anti-tumor pharmaceutical applications.

## 2 Inhibition of cancer cell proliferation

### 2.1 Induction of cell cycle arrest

The cell cycle is a highly conserved and precisely controlled process that consists of four distinct, ordered phases (G0/G1, S, G2, and M). Uncontrolled cell proliferation is a key factor in tumorigenesis and tumor progression. Cell cycle regulators such as cyclin-dependent kinase 2 (CDK2), CDK4, and cyclin D1 play an important role in this process. CDKs are key regulatory enzymes involved in cell proliferation by regulating cell cycle checkpoints and transcriptional events in response to extracellular and intracellular signals (Ding et al., 2020). Cyclin D1 is a key regulator of the cell cycle and plays a central role in the pathogenesis of cancer by determining uncontrolled cell proliferation (Fuste et al., 2016). Esculetin (10–100 μM) has been demonstrated to exert antiproliferative effects on osteosarcoma LM8 cells by inducing G1 cell cycle arrest. The mechanism of action appears to involve the suppression of CDK4 and cyclin D1 expression (Kimura and Sumiyoshi, 2015). Another study observed that esculetin induced G0/G1 cell cycle arrest in human leukemia cells (HL-60), which was accompanied by the suppression of cyclin D1, cyclin D3, CDK2, and CDK4 (Wang et al., 2019). Additionally, it was shown that esculetin induces cell cycle arrest at the G0/G1 phase in human colon cancer LoVo cells and that the protein expression of cyclin D1 is decreased after treatment with esculetin (Choi et al., 2019). Fraxetin has been demonstrated to induce tumor cell cycle arrest in various cell lines, including Huh7 and Hep3B hepatocellular carcinoma cell lines, where it causes cells to arrest at the G0/G1 phase (Song et al., 2021). Furthermore, fraxetin was shown to effectively induce cell cycle arrest at the G0/G1 phase by downregulating the expression of the G1/S transition regulatory proteins cyclin D1, CDK4, and CDK6 in human non-small cell lung cancer cell lines (HCC827 and H1650) (Zhang G. et al., 2019).

### 2.2 Enhancement of p53, p21, and p27 expression

p53 is a primary tumor suppressor gene that functions to inhibit proliferation and eliminate abnormal cells. p53 regulates a variety of genes and activates various responses, including cell cycle arrest and apoptosis (Soussi, 2000). p21 is a potent CDK-binding inhibitor that is transcriptionally controlled by p53 pathways and is a key negative regulator of the cell cycle (Jassim et al., 2021). p27 has the function of binding to and inhibiting cyclin-CDK, leading to cell cycle arrest (Razavipour et al., 2020). Esculin has been reported to reduce the viability and proliferation of MDA-MB-231 triple-negative breast cancer cell lines in a dose- and time-dependent manner. In addition, the levels of p53 and p21 mRNAs and proteins were increased in a concentration-dependent manner after treatment with esculin (Mo et al., 2020). In human colon cancer LoVo cells, esculetin treatment inhibited LoVo cell proliferation and was accompanied by increased expression of p53, p21, and p27 proteins (Choi et al., 2019). Additionally, it was proposed that esculetin inhibits the proliferation of human prostate cancer cell lines (PC3, DU145, and LNCaP) by increasing the expression of p53, p21, and p27 (Turkekul et al., 2018).

### 2.3 Suppression of Ki-67 and PCNA expression

The antigen Ki-67, also known as Ki-67 or Marker of Proliferation Ki-67 (MKI67), is a human protein encoded by the MKI67 gene (Davey et al., 2021). Ki-67 protein has been widely used as a proliferation marker for human tumor cells, and its expression is closely related to tumor prognosis (Louis et al., 2023). Proliferating cell nuclear antigen (PCNA) has been found in the nuclei of yeast, plant, and animal cells that are in the process of cell division, suggesting that it plays a role in regulating the cell cycle and/or DNA replication (Strzalka and Ziemienowicz, 2011). PCNA is considered essential for DNA replication in cancer cells and has also been implicated in tumor invasion (Wang et al., 2022). Esculetin was reported to inhibit human colorectal cancer HCT-116 cell proliferation by decreasing the expression of the proliferation-related proteins Ki-67 and PCNA (Yan et al., 2019). *In vivo* experiments in the HCT-116 subcutaneous tumor-bearing model also demonstrated a significant decrease in the expression of Ki-67 and PCNA following treatment with esculetin. In a xenograft model of colon cancer, treatment with esculetin at 20 mg/kg and 100 mg/kg significantly reduced tumor size by 44% and 64%, respectively. Furthermore, the expression of Ki-67 was suppressed by esculetin treatment in both colon cancer cells and xenograft tumor tissues (Lee et al., 2013). In human pancreatic cancer cell lines (PANC-1 and Patu8988), fraxetin was demonstrated to inhibit cell proliferation and reduce the expression of Ki-67 (Guo et al., 2021). Additionally, fraxetin was demonstrated to inhibit proliferation and reduce the protein expression of Ki-67 in human glioblastoma U251 cells (Qu et al., 2021) (Table 1).

**TABLE 1:** Inhibition of cancer cell proliferation.

Metabolite	Mechanism	Research type	Cancer	Cell line	Dose/route of administration	Ref
Esculetin	CDK2, CDK4, cyclin D1, and cyclin D3↓	*In vitro*	osteosarcoma, leukemia, and colon cancer	LM8, HL-60, and LoVo	10, 50, 75, and 100 μM (LM8), 20 μM (HL-60), 200, 400, and 600 μM (LoVo)	Kimura and Sumiyoshi, (2015), Wang et al. (2019), Choi et al. (2019)
	p53, p21, and p27↑	*In vitro*	colon cancer and prostate cancer	LoVo and PC3	200, 400, and 600 μM (LoVo), 75 and 150 μM (PC3)	Choi et al. (2019), Turkekul et al. (2018)
	Ki-67 and PCNA↓	*In vivo* *and* *in vitro*	colon cancer	HCT-116	20 μg/mL (HCT-116), 50 mg/kg (intragastric administration), 20 and 100 mg/kg (intraperitoneal injection)	Yan et al. (2019), Lee et al. (2013)
Esculin	p53 and p21↑	*In vitro*	triple-negative breast cancer	MDA-MB-231	225, 450, and 900 μmol/L	Mo et al. (2020)
Fraxetin	cyclin D1, CDK4, and CDK6↓	*In vitro*	non-small cell lung cancer	HCC827 and H1650	25, 50, and 100 μM	Zhang et al. (2019b)
	Ki-67↓	*In vitro*	pancreatic cancer and glioblastoma	PANC-1, Patu8988, and U251	50 and 100 μM (PANC-1 and Patu8988), 100 and 200 μM (U251)	Guo et al. (2021), Qu et al. (2021)

## 3 Induction of cancer cell apoptosis

### 3.1 Induction of the mitochondrial apoptotic pathway

Esculetin has been shown to induce apoptosis of gastric cancer cell lines (MGC-803, HGC-27, and BGC-823) in a dose-dependent manner with lower toxicity against normal gastric epithelial cells (Wang G. et al., 2017). A mechanistic study revealed that esculetin induced apoptosis in MGC-803 cells by triggering the activation of the mitochondrial apoptotic pathway, increasing cytochrome c release from mitochondria, and activating caspase-3 and caspase-9 activity. In addition, esculetin dose-dependently inhibited the proliferation of human colon cancer LoVo cells and activated caspase-3, caspase-7, and caspase-9 (Choi et al., 2019). In human A549 lung cancer cells, esculin was shown to increase the activity of caspase-3 and caspase-9 (Wang, 2015). It has been demonstrated that esculetin is capable of inducing apoptosis in human leukemia HL-60 cells in a dose-dependent manner. Furthermore, esculetin treatment enhanced the cleavage of caspase-3 and caspase-9 and induced the release of cytochrome c in HL-60 cells (Wang et al., 2019). In another study, esculetin treatment led to Ca^2+^ influx and induced Ca^2+^ release from the endoplasmic reticulum in human breast cancer ZR-75-1 cells, thereby activating the Ca^2+^-associated mitochondrial apoptotic pathway (Chang et al., 2016). Fraxetin was also found to promote apoptosis by the mitochondrial pathway in tumor cells. It was reported that fraxetin inhibited proliferation and induced apoptosis in human endometrial cancer RL95-2 cells. Furthermore, the treatment with fraxetin resulted in a reduction of the mitochondrial membrane potential and an increased expression of caspase-3 and caspase-9 (Xu et al., 2020). Caspase-3 and caspase-9 are both members of the cysteine protease family. Caspase-9 is an initiator caspase; activated caspase-9 cleaves downstream caspases such as caspase-3, caspase-6, and caspase-7 (Kuida, 2000). Caspase-3 is the main executioner of apoptosis; it is cleaved and activated during the early stages of apoptosis to execute apoptosis by cleaving targeted cellular proteins (Eskandari and Eaves, 2022).

### 3.2 Regulation of the Bcl-2 protein family

Members of the B cell lymphoma-2 (Bcl-2) protein family are key regulators with pro- or anti-apoptotic activities. By affecting mitochondrial membrane permeability changes, the pro- and anti-apoptotic proteins of the Bcl-2 family are involved in the regulation of apoptosis (Qian et al., 2022). Bcl-2 is a well-known anti-apoptotic protein that has been shown to promote carcinogenesis by resisting cell death (Warren et al., 2019). B-cell lymphoma-extra large (Bcl-xL) is also a member of the Bcl-2 family of anti-apoptotic proteins. Bcl-2-associated X protein (Bax) is a pro-apoptotic protein that can commit a cell to programmed death by permeabilizing the outer mitochondrial membrane and subsequently initiating the caspase cascade (Edlich, 2018). Bcl-2 antagonist/killer (Bak) is another pro-apoptotic factor, similar to Bax, that promotes apoptosis and increases susceptibility to apoptosis under appropriate stimulation (Qian et al., 2022). Esculetin was shown to exert a significant inhibitory effect on the viability of hepatocellular carcinoma SMMC-7721 cells in a dose- and time-dependent manner. And esculetin was able to reduce the weight of the tumors in a xenograft model of hepatocellular carcinoma (Wang et al., 2015). Mechanistic studies demonstrated that esculetin induced a mitochondrial-mediated apoptotic pathway *in vivo* and *in vitro*, increased Bax protein expression, decreased Bcl-2 protein expression, and caused loss of mitochondrial membrane potential. In oral squamous cell carcinoma cell lines (HN22 and HSC4), esculetin was also shown to induce apoptosis by upregulating Bax and downregulating Bcl-xL (Jeon et al., 2016). Similarly, fraxetin was found to exert regulatory effects on Bcl-2 family members. It was demonstrated that fraxetin suppressed cell viability and induced apoptotic cell death in HT-29 and HCT-116 colon cancer cell lines. Furthermore, fraxetin treatment resulted in an increase in the expression of Bax and Bak and a decrease in the expression of Bcl-2 and Bcl-xL in HT-29 and HCT-116 cells. (Lee et al., 2022). Additionally, fraxetin was shown to upregulate the expression of Bax and downregulate the expression of Bcl-2 in human non-small cell lung cancer cell lines (HCC827 and H1650), thereby inducing apoptosis (Zhang Y. et al., 2019).

### 3.3 Induction of reactive oxygen species

Reactive oxygen species (ROS) are a group of highly reactive molecules that play a central role in cell signaling and regulation of apoptosis pathways. It has been demonstrated that ROS are intimately associated with the apoptotic activation of the mitochondrial pathway. Moreover, there is a correlation between ROS and death receptor-induced apoptosis, as well as endoplasmic reticulum stress-induced apoptosis (Redza-Dutordoir and Averill-Bates, 2016). It was reported that esculetin stimulated the generation of intracellular ROS and induced apoptosis in HT-29 colorectal cancer cells (Kim et al., 2015). Pan et al. reported that esculetin inhibited the growth of human gastric cancer cell lines (SGC-7901, MGC-803, and BGC-823) in a dose- and time-dependent manner and reduced the viability of gastric cancer cells by inducing apoptosis. Moreover, it was proposed that elevated intracellular ROS levels were a pivotal factor in esculetin-mediated cell death (Pan et al., 2015). Fraxetin treatment has been shown to promote apoptosis and induce ROS generation in colon cancer cell lines (HT-29 and HCT-116) (Lee et al., 2022), hepatocellular carcinoma cell lines (Huh7 and Hep3B) (Song et al., 2021), and pancreatic cancer cell lines (PANC-1 and Patu8988) (Guo et al., 2021) (Table 2).

**TABLE 2:** Induction of cancer cell apoptosis.

Metabolite	Mechanism	Research type	Cancer	Cell line	Dose/route of administration	Ref
Esculetin	cytochrome c↑	*In vitro*	gastric cancer and leukemia	MGC-803 and HL-60	425, 850, and 1,700 μM (MGC-803), 20 μM (HL-60)	Wang et al. (2017a), Wang et al. (2019)
	caspase-3, caspase-7, and caspase-9↑	*In vitro*	gastric cancer, colon cancer, and leukemia	MGC-803, LoVo, and HL-60	425, 850, and 1,700 μM (MGC-803), 200, 400, and 600 μM (LoVo), 20 μM (HL-60)	Wang et al. (2017b), Choi et al. (2019), Wang et al. (2019)
	Ca^2+^↑	*In vitro*	breast cancer	ZR-75–1	20, 40, and 60 μM	Chang et al. (2016)
	Bax↑ Bcl-2, Bcl-xL↓	*In vivo* and *in vitro*	hepatocellular carcinoma and oral squamous cell carcinoma	SMMC-7721, HN22, and HSC2	200, 400, and 700 mg/kg (intraperitoneal injection), 1.12, 2.24, 4.48 mM (SMMC-7721), 5, 10, and 20 mg/mL (HN22 and HSC2)	Wang et al. (2015), Jeon et al. (2016)
	ROS↑	*In vitro*	colon cancer and gastric cancer	HT-29 and MGC-803	55 μg/mL (HT-29), 12.5, 25, and 50 μM (MGC-803)	Kim et al. (2015), Pan et al. (2015)
Esculin	caspase-3 and caspase-9↑	*In vitro*	lung cancer	A549	0.4, 0.8, and 1.6 mmol/L	Wang (2015)
Fraxetin	caspase-3 and caspase-9↑	*In vitro*	endometrial cancer	RL95-2	5, 10, 20, and 40 μM	Xu et al. (2020)
	Bax and Bak↑ Bcl-2 and Bcl-xL↓	*In vitro*	colon cancer and non-small cell lung cancer	HCT-116, HT-29, HCC827, and H1650	20, 50, and 100 μM (HCT-116 and HT-29), 25, 50, and 100 μM (HCC827 and H1650)	Lee et al. (2022), Zhang et al. (2019a)
	ROS↑	*In vitro*	colon cancer, hepatocellular carcinoma, and pancreatic cancer	HCT-116, HT-29, Huh7, Hep3B, PANC-1, and Patu8988	20, 50, 100, and 200 μM (HCT-116 and HT-29), 5, 10, 20, and 50 μM (Huh7 and Hep3B), 50 and 100 μM (PANC-1 and Patu8988)	Lee et al. (2022), Song et al. (2021), Guo et al. (2021)

## 4 Suppression of cancer cell invasion and migration

### 4.1 Regulation of epithelial-mesenchymal transition

The epithelial-to-mesenchymal transition (EMT) plays a pivotal role in both developmental processes and the progression of cancer. EMT enables solid tumors to become more malignant, increasing their invasiveness and metastatic activity. Events that occur during EMT include the loss of adherens junctions, the downregulation of epithelial-specific markers (e.g., cytokeratin and E-cadherin), and the upregulation of mesenchymal markers (e.g., fibronectin, N-cadherin, and vimentin) (Zhang and Weinberg, 2018). It was reported that esculetin inhibited the migration and EMT of colorectal cancer HCT-116 cells by downregulating EMT-related proteins N-cadherin, vimentin, and fibronectin (Yan et al., 2019). Xu et al. demonstrated that fraxetin suppressed the expression of EMT-associated markers N-cadherin, snail, and vimentin and increased the expression of E-cadherin in ovarian cancer cell lines (SKOV3 and SW626) (Xu et al., 2023). Furthermore, fraxetin treatment was also found to suppress the invasion and migration of pancreatic cancer PANC-1 and Patu8988 cells by regulating the Slug-E-cadherin axis-dependent EMT process (Guo et al., 2021).

### 4.2 Suppression of matrix metalloproteinase expression

Matrix metalloproteinases (MMPs) are a family of zinc-dependent proteolytic endopeptidases with extracellular matrix remodeling and degradation properties and have long been implicated in cancer initiation, tumor growth, and metastasis (Knapinska et al., 2017). Esculetin was found to suppress the expression of MMP-2 and MMP-7 in HCT-116 cells, thereby inhibiting cell migration. *In vivo* experiments of the HCT-116 subcutaneous tumor-bearing model also demonstrated that esculetin treatment significantly decreased the expression of MMP-2 and MMP-7 (Yan et al., 2019). Moreover, esculetin was shown to suppress the expression of MMP-2 in osteosarcoma LM8 cells (Kimura and Sumiyoshi, 2015). Qu and colleagues observed that fraxetin inhibited the invasion and migration of human glioblastoma U251 cells *in vitro* and downregulated the expression levels of MMP-2 and MMP-9 (Qu et al., 2021) (Table 3).

**TABLE 3:** Suppression of cancer cell invasion and migration.

Metabolite	Mechanism	Research type	Cancer	Cell line	Dose/route of administration	Ref
Esculetin	N-cadherin, vimentin, and fibronectin↓	*In vitro*	colorectal cancer	HCT-116	20 μg/mL	Yan et al. (2019)
	MMP-2 and MMP-7↓	*In vivo* and *in vitro*	colorectal cancer and osteosarcoma	HCT-116 and LM8	50 mg/kg (intragastric administration), 20 μg/mL (HCT-116), 10, 50, 75, and 100 μM (LM8)	Yan et al. (2019), Kimura and Sumiyoshi (2015)
Fraxetin	N-cadherin, snail, and vimentin↓ E-cadherin↑	*In vitro*	ovarian cancer and pancreatic cancer	SKOV3, SW626, PANC-1, and Patu8988	40, 60, and 80 μmol/L (SKOV3 and SW626), 50 and 100 μM (PANC-1 and Patu8988)	Xu et al. (2023), Guo et al. (2021)
	MMP-2 and MMP-9↓	*In vitro*	glioblastoma	U251	100 and 200 μM	Qu et al. (2021)

## 5 Regulation of tumor-associated intracellular signaling pathways

### 5.1 Inhibition of the PI3K/Akt signaling pathway

The phosphoinositide 3-kinase (PI3K)/protein kinase B (Akt) signaling pathway is one of the most frequently overactivated intracellular pathways in several human cancers (Rascio et al., 2021). The PI3K/Akt pathway plays a multitude of roles in the onset and progression of cancer, including the promotion of cancer cell proliferation and migration as well as the prevention of apoptosis. One study reported that application of esculin to human glioblastoma T98G cells caused apoptosis in approximately 15% of the cells. It was further suggested that this apoptosis-inducing effect may be associated with an increase in the expression of caspase-3 and a decrease in the expression of PI3K (Sumorek-Wiadro et al., 2020). In gastric cancer MGC-803 cells, esculetin was found to promote apoptosis by downregulating the PI3K/Akt signaling pathway. Furthermore, the inhibitory effect of esculetin on the PI3K/Akt pathway was also observed in a nude mouse model subcutaneously inoculated with MGC-803 cells (Wang Y. et al., 2017). Ma et al. reported that fraxetin significantly inhibited the viability, proliferation, migration, and invasion of prostate cancer DU145 cells and induced cell apoptosis in a concentration-dependent manner (Ma et al., 2022). Moreover, the expression of polo-like kinase 4 (PLK4), p-PI3K, and p-Akt was found to be decreased by fraxetin treatment. The researchers postulated that fraxetin may act as a tumor suppressor in prostate cancer by inhibiting PLK4 expression, which in turn inactivates the PI3K/Akt signaling pathway.

### 5.2 Inhibition of the MAPK/ERK pathway

Mitogen-activated protein kinase (MAPK) cascades represent a fundamental signaling pathway that regulates a multitude of cellular processes, including proliferation, differentiation, apoptosis, and stress responses (Guo et al., 2020). Extracellular signal-regulated kinase 1/2 (ERK1/2) is a member of the MAPK family, which plays a pivotal role in tumor proliferation, invasion, and metastasis (Ali et al., 2022). The activation of the MAPK/ERK pathway has been demonstrated to promote proliferation and have an anti-apoptotic effect (Bahar et al., 2023). Wang et al. demonstrated that esculetin inhibited the proliferation of human leukemia HL-60 cells in a dose-dependent manner, inducing apoptosis and autophagy in HL-60 cells (Wang et al., 2019). Moreover, esculetin was observed to block the ERK signaling pathway in HL-60 cells in a concentration-dependent manner. Another study demonstrated that esculetin inhibited the cell viability of human leukemia U937 cells by inducing apoptosis and selectively inhibiting the phosphorylation of ERK (Lin et al., 2009). Fraxetin was shown to inhibit the proliferation of MCF-7 breast cancer cells in a dose- and time-dependent manner, as well as induce cell cycle arrest at the G0/G1 phase. Furthermore, it was demonstrated that fraxetin treatment resulted in a reduction in the expression of p-ERK1/2 in MCF-7 cells, indicating that fraxetin may exert an anti-tumor effect through the MAPK/ERK1/2 signaling pathway (Huo et al., 2013).

### 5.3 Inhibition of the JAK/STAT3 pathway

The signal transducer and activator of transcription 3 (STAT3) pathway is intimately linked to the tumor microenvironment, tumor growth, and metastasis. Excessive STAT3 activation in cancer cells and the tumor microenvironment can be viewed as a neoplastic mimic of an inflammation-driven repair response that collectively drives tumor progression (Huynh et al., 2019). It was demonstrated that esculetin significantly inhibited the proliferation, migration, and invasion of laryngeal cancer Hep-2 cells and significantly inhibited the phosphorylation of Janus kinase 1 (JAK1), JAK2, and STAT3 in Hep-2 cells. Furthermore, esculetin treatment was observed to reduce tumor growth and tumor weight in a dose-dependent manner in laryngeal cancer xenograft mice (Zhang G. et al., 2019). The potential mechanism of action may be attributed to its inhibition of the JAK/STAT3 signaling pathway. Qu et al. found that fraxetin inhibited the proliferation, invasion, and migration of human glioblastoma U251 cells *in vitro* and significantly reduced tumor volume and weight *in vivo*. Moreover, fraxetin treatment resulted in a reduction in the expression of p-JAK2 and p-STAT3 in both U251 cells and glioma xenograft mice (Qu et al., 2021). In human pancreatic cancer cell lines (PANC-1 and Patu8988), fraxetin was demonstrated to inhibit cell proliferation and induce mitochondrial-dependent apoptosis. It was demonstrated that fraxetin prevents the formation of STAT3 homodimers through intimate contact with the SH2 domain of STAT3. Thus, fraxetin inhibited STAT3 phosphorylation and blocked the activation of downstream STAT3 signaling pathways (Guo et al., 2021).

### 5.4 Inhibition of the Wnt/β-catenin pathway

The Wnt/β-catenin signaling pathway has been extensively implicated in the pathogenesis of cancers. Aberrant activation of the Wnt/β-catenin signaling pathway is closely associated with an increased prevalence of cancer, the advancement of malignant progression, the development of a poor prognosis, and even an increase in cancer-related mortality (Yu et al., 2021). Esculetin has been demonstrated to suppress the activity of the Wnt/β-catenin signaling pathway in human colorectal cancer cells (HCT116 and SW480), thereby exerting anti-proliferative effects (Kim et al., 2018). Fan et al. reported that esculetin dose- and time-dependently reduced cell viability and decreased the mRNA and protein levels of β-catenin in human hepatocellular carcinoma SMMC-7721 cell lines (Fan et al., 2017). These findings suggest that esculetin may play an anti-tumor role by inhibiting the Wnt/β-catenin signaling pathway (Table 4).

**TABLE 4:** Regulation of tumor-associated intracellular signaling pathways.

Metabolite	Mechanism	Research type	Cancer	Cell line	Dose/route of administration	Ref
Esculetin	PI3K/Akt↓	*In vivo* and *in vitro*	gastric cancer	MGC-803	850 μM, 50 and 100 mg/kg (intragastric administration)	Wang et al. (2017a)
	ERK↓	*In vitro*	leukemia	HL-60 and U937	5, 10, and 20 μM (HL-60), 50 and100 μM (U937)	Wang et al. (2019), Lin et al. (2009)
	p-JAK1, p-JAK2, and p-STAT3↓	*In vitro*	laryngeal cancer	HCC827 and H1650	12.5, 25, 50 and 100 μM	Zhang et al. (2019b)
	Wnt/β-catenin↓	*In vitro*	colorectal cancer and hepatocellular carcinoma	HCT-116, SW480, and SMMC-7721	20, 40 and 60 μM (HCT-116 and SW480), 100 and 300 μmol/L (SMMC-7721)	Kim et al. (2018), Fan et al. (2017)
Esculin	PI3K↓	*In vitro*	glioblastoma	MOGGCCM and T98G	200 μM	Sumorek-Wiadro et al. (2020)
Fraxetin	PLK4↓ PI3K/Akt↓	*In vitro*	prostate cancer	DU145	10, 20 and 40 μM	Ma et al. (2022)
	p-ERK1/2↓	*In vitro*	breast cancer	MCF-7	40 μmol/L	Huo et al. (2013)
	p-JAK2 and p-STAT3↓	*In vivo* and *in vitro*	Glioblastoma and pancreatic cancer	U251, PANC-1, and Patu8988	100 and 200 μM (U251), 50 and 100 μM (PANC-1 and Patu8988), 25 mg/kg (intragastric administration)	Qu et al. (2021), Guo et al. (2021)

## 6 Conclusion and perspectives

**FIGURE 2:**
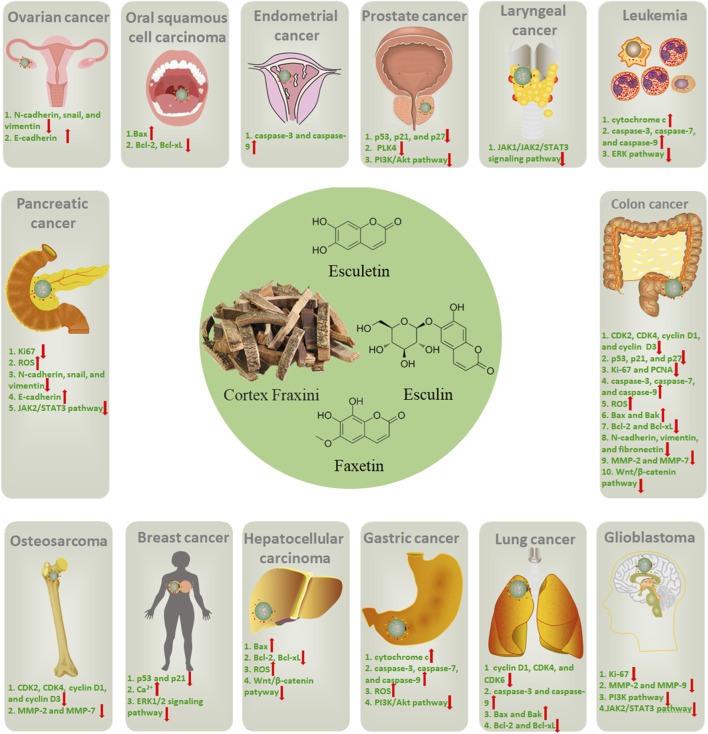
The potential roles of Cortex Fraxini in different types of cancer.

In the current review, it has been demonstrated that the active metabolites in Cortex Fraxini, such as esculin, esculetin, and fraxetin, have the capacity to inhibit the growth of many different types of cancer, including osteosarcoma, leukemia, colon cancer, prostate cancer, breast cancer, glioblastoma, gastric cancer, lung cancer, hepatocellular carcinoma, and oral squamous cell carcinoma (Figure 2). These active metabolites have been shown to inhibit tumor cell growth through induction of cell cycle arrest, upregulation of p53, p21, and p27, and inhibition of Ki-67 and PCNA expression. Furthermore, it was proposed that they could promote apoptosis in tumor cells by inducing the mitochondrial apoptotic pathway, regulating the Bcl-2 family of proteins, and inducing the production of ROS. Additionally, they were demonstrated to inhibit tumor cell invasion and migration by modulating EMT and inhibiting MMP expression. Furthermore, these active metabolites could regulate several tumor-associated signaling pathways, including the PI3K/Akt, MAPK/ERK, JAK/STAT3, and Wnt/β-catenin pathways. In summary, a large number of studies have been conducted with the aim of elucidating the anti-tumor pharmacological effects and mechanisms of Cortex Fraxini and its active metabolites, including esculin, esculetin, and fraxetin. In view of their pro-apoptotic and anti-proliferative properties *in vitro* and *in vivo*, Cortex Fraxini and its active metabolites can be considered potential candidates for the treatment of tumors.

Although numerous studies have been conducted to investigate the anti-tumor pharmacological effects of the active metabolites of Cortex Fraxini, there are still some deficiencies that require further research and improvement in the future. Firstly, given that many of the anti-tumor effects of Cortex Fraxini are only phenotypic variations, in-depth research to explore the complex mechanistic aspects should be a priority. Secondly, the majority of studies to date have employed *in vitro* experiments as the primary methodology, with a paucity of further proof from *in vivo* studies. Therefore, the utilization of tumor animal models should be enhanced in future studies to verify the aforementioned anti-tumor mechanisms. Thirdly, although coumarins are the primary active metabolites of Cortex Fraxini, the roles of its other metabolites, including lignans, secoiridoids, phenylethanol glycosides, flavonoids, and triterpenoids, in its anti-tumor activities require further elucidation. Fourthly, how to improve the bioavailability of the metabolites is also a future research direction. To address this challenge, some researchers have constructed a nanostructured lipid carrier loaded with esculetin, which improves its oral bioavailability by 1.7 times, thus enhancing its pharmacological role (Shi et al., 2023). Another study developed an esculetin-loaded mixed micellar delivery system that specifically increased the biological availability of esculetin by 3.06 times (Li et al., 2023). Fifthly, according to existing reports, the active metabolites of Cortex Fraxini have inhibitory effects on many types of tumors, but whether their anti-tumor activity differs among different tumors deserves further investigation.

## Abbreviations

Akt, protein kinase B; Bak, Bcl-2 antagonist/killer; Bax, Bcl-2-associated X protein; Bcl-2, B cell lymphoma-2; Bcl-xL, B-cell lymphoma-extra large; CDK, cyclin-dependent kinase; EMT, epithelial-to-mesenchymal transition; ERK, extracellular signal-regulated kinase; JAK, Janus kinase; MAPK, mitogen-activated protein kinase; MEK, mitogen-activated protein kinase; MMP, matrix metalloproteinase; PCNA, proliferating cell nuclear antigen; PI3K, phosphoinositide 3-kinase; PLK4, polo-like kinase 4; Ras, rat sarcoma; ROS, reactive oxygen species; STAT3, signal transducer and activator of transcription 3; TYK2, tyrosine kinase 2.
